# Assessment of Clinical and Patient-Centered Outcomes in Nonsurgical Periodontal Therapy

**DOI:** 10.7759/cureus.56464

**Published:** 2024-03-19

**Authors:** Vyshnavi B Sindhusha, Arvina Rajasekar

**Affiliations:** 1 Periodontics, Saveetha Dental College and Hospitals, Saveetha Institute of Medical and Technical Sciences, Saveetha University, Chennai, IND

**Keywords:** clinical measures, quality of life (qol), patient centered outcomes, periodontal therapy, oral health

## Abstract

Aim

The study was conducted to assess the clinical and patient-centered outcomes among the patients who had undergone nonsurgical periodontal therapy (NSPT).

Methodology

The participants for this study were 40 individuals with generalized chronic periodontitis. Numerous clinical parameters including clinical attachment level (CAL), probing pocket depth (PPD), plaque index (PI), and gingival index (GI) were evaluated along with the administration of a customized questionnaire before and after three months of therapy to evaluate patient-centered outcomes.

Results

Clinical parameters showed significant (p < 0.05) improvement post-NSPT. There was 100% satisfaction in few patient-centered outcomes such as bleeding gums, bad breath, food entrapment, and mobility.

Conclusion

A significant improvement in the clinical parameters does not guarantee improvement in patient-centered outcomes. Achieving the improvement in patient-centered outcomes can improve the overall quality of life (QOL), marking this a holistic treatment.

## Introduction

Periodontal diseases like gingivitis and periodontitis are a significant health and economic burden as they affect a large portion of the global population. Periodontitis can be described as a chronic inflammatory condition associated with numerous systematic disorders like coronary artery disease and diabetes [1]. Gingivitis happens with gingiva inflammation due to the accumulation of plaque and food debris. But if not treated it could result in periodontitis that can destroy the alveolar bone and periodontal ligament. This progression occurs due to the body's immune response to bacteria in the plaque resulting in chronic inflammation and ultimately in tooth loss [2]. Clinically, periodontitis is categorized with periodontal pockets and loss of connective tissue attachment, and radiographs reveal the extent of bone loss around the affected teeth [3].

Nonsurgical periodontal therapy (NSPT) is the gold standard procedure to treat periodontitis, and the primary aim of the treatment is to eliminate the etiology of the disease and to promote healing and reattachment of the periodontal tissues [4]. This procedure involves the thorough removal of plaque and inner lining of diseased gingiva within the periodontal pocket along with planning the root surfaces to remove the diseased cementum to restore the biocompatibility of the root surface and promote the new attachment of gingival tissue [5]. While all the clinical parameters such as bleeding on probing (BOP), probing pocket depth (PPD), and clinical attachment loss (CAL) provide information about the activity of the periodontal disease, they will not fully capture the effect of treatment on the patient's overall quality of life and oral health. However, a successful NSPT should measure patient-centered outcomes as well, which include gingival appearance, loosening of teeth, bad breath, food lodgment, masticatory function, and so on [6]. A workshop conducted in 2003 identified patient-based outcomes (PBO). The patient-centered outcomes have gained more recognition as they focus on understanding and addressing the individual patient's needs and preferences [7]. Several questionnaires and indices have been developed to measure PBO for assessing the effect of oral health on overall quality of life such as “Oral Health Assessment Index” (GOHAI), Dental Impact Profile, Oral Impacts on “Daily Performances, Dental Impact on Daily Living” (DIDL), “Oral Health Impact Profile” (OHIP), and Child Perception Questionnaire [8].

In literature, various studies have utilized oral health measurement scales and questionnaires to evaluate patient-centered outcomes in different dental procedures and interventions such as implant-supported fixed dentures [9], management of dental caries [10], and surgical periodontal therapy [11]. In the field of periodontology, the patient-centered outcomes have been assessed only for surgical periodontal therapy in various studies, but the patient-centered outcomes for NSPT were not evaluated to date.

The present study in this context aimed to assess the clinical and patient-centered outcomes among the patients who had undergone NSPT.

## Materials and methods

Study population

**Figure 1:**
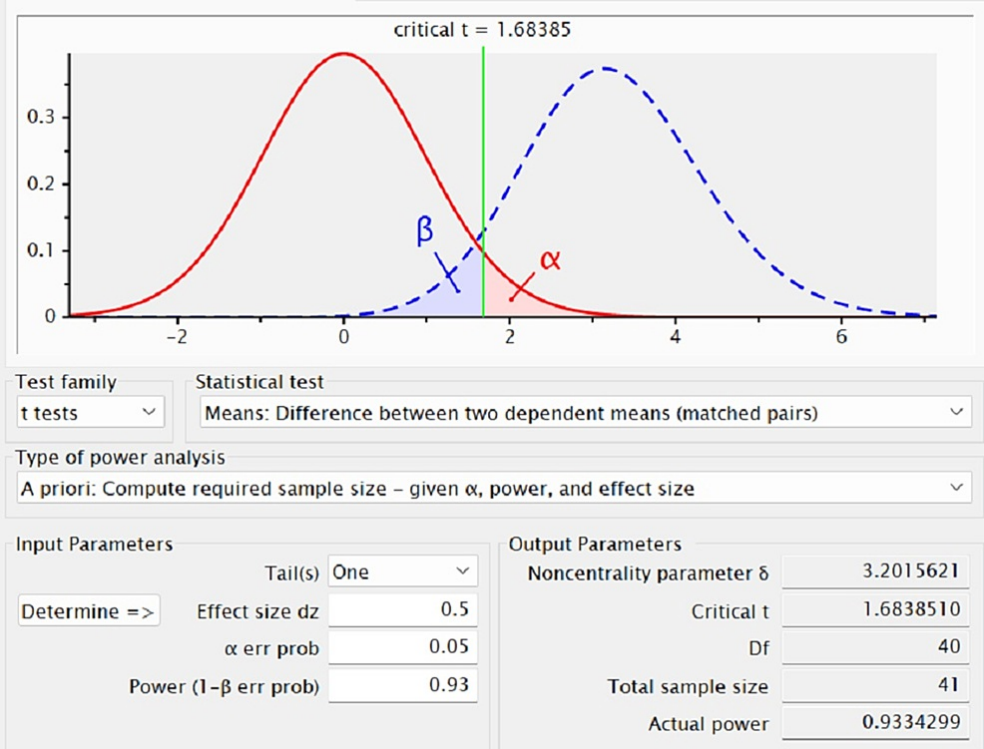
Calculation of the study population using G*Power software G* power software was used to calculate the sample size where critical t (number of standard deviations from null mean) and degrees of freedom (df) along with noncentrality parameter gamma (δ) are the outcome parameters to obtain sample size.

A total of 40 subjects, 20 females and 20 males, in the 25 to 50 years age group diagnosed with generalized chronic periodontitis and aggressive periodontitis with a probing pocket depth of 4 to 5 mm in the Department of Periodontics, Saveetha Dental College and Hospitals, Chennai, were considered as study participants after attaining proper informed consent. The research was approved by the institutional review board (SRB/SDC/PERIO-2104/23/011). The sample size was determined with the help of G*Power statistical software (Figure 1).

Inclusion criteria

Patients who were devoid of systemic conditions, nonsmokers, and those with CAL < 5 mm and PPD < 5 mm were included as study subjects.

Exclusion criteria

Patients with the habit of smoking or using tobacco products, who underwent periodontal therapy in the last six months, those with systemic diseases, and those who were under long-term medications were excluded from the study.

NSPT

The treatment procedure involved supragingival scaling using a piezoelectric ultrasonic device followed by a manual root planning under local anesthesia for a time period of half an hour performed by a calibrated periodontist. The root planning was done using conventional Gracey curettes with specific numbers (1/2, 3/4, 11/12, 13/14, Hu-Friedy). As part of the study protocol, the subjects received oral hygiene instructions (OHI).

Parameters

**Figure 2:**
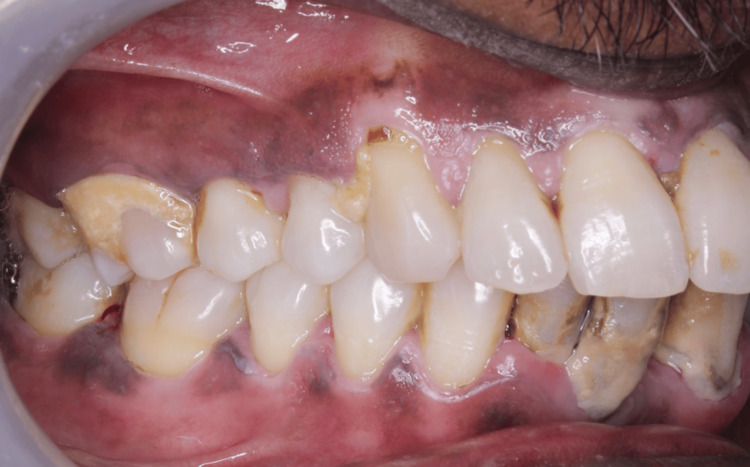
At baseline (before NSPT) NSPT: nonsurgical periodontal therapy The above image shows the plaque and calculus along with mild inflammation in the interdental papilla before performing NSPT.

**Figure 3:**
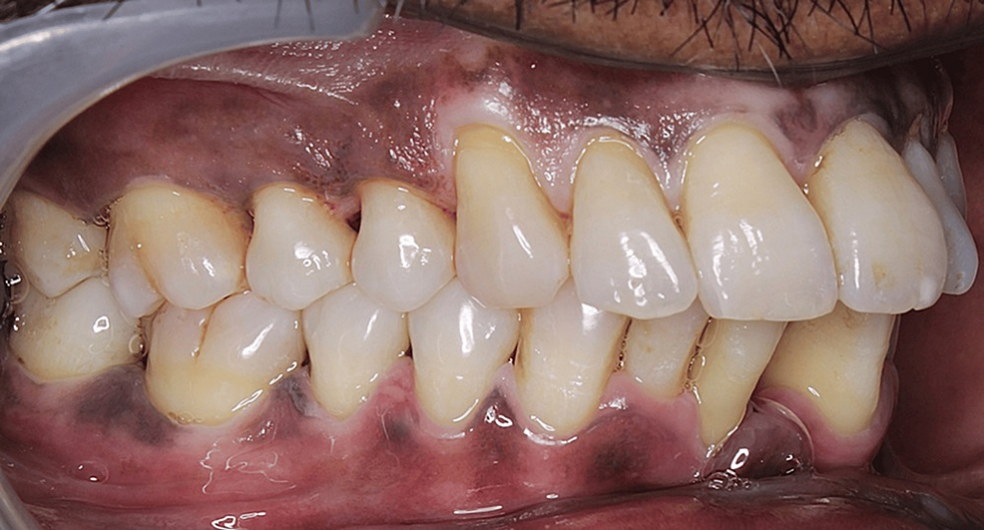
Three-month recall visit (post-NSPT) NSPT: nonsurgical periodontal therapy The above image shows the reduced plaque and inflammation along with the accentuated gingival stippling in the interdental papilla during a three-month recall visit after performing NSPT.

All the study participants were scored for clinical parameters (CAL, PPD, PI, and GI) at baseline (Figure 2) and three months (Figure 3).

A customized questionnaire was prepared with relevant questions to evaluate the patients' perceptions with the help of dental impact on daily living (DIDL) questionnaire prior to NSPT (baseline) and three months post-therapy.

Statistical analysis

Data obtained was analyzed with IBM SPSS Statistics for Windows, Version 23 (Released 2015; IBM Corp., Armonk, New York, United States). The standard deviation and mean were calculated for all clinical parameters such as gingival index (GI), plaque index (PI), PPD, and CAL. Paired t-test was done for comparing these parameters between baseline and at three months. Wilcoxon signed-rank test was done to test the statistical significance of the questionnaire between baseline and at three months.

## Results

Results showed that clinical parameters (GI, PI, PPD, and CAL) have markedly improved at three months of NSPT with p < 0.05 (Table 1).

**Table 1:** Comparison of the clinical parameters The table presents the mean± standard deviation along with the p-values of all the clinical parameters such as gingival index (GI), plaque index (PI), probing pocket depth (PPD), and clinical attachment loss (CAL) at baseline and at three-month post-operative review of nonsurgical periodontal therapy (NSPT). Paired t-test was performed (p < 0.05).

Clinical parameters	Mean ± standard deviation		p-value
	Baseline	3 months	
Gingival index (GI)	2.53±0.27	0.47±0.21	0.05
Plaque index (PI)	2.76±0.47	0.35±0.18	0.03
Probing pocket depth (PPD)	4.32±0.29	1.85±0.12	0.01
Clinical attachment level (CAL)	5.76±1.27	2.28±1.09	0.02

The customized questionnaire which was prepared using the DIDL questionnaire was scored pre- and at three months post-NSPT (Table 2).

**Table 2:** Comparison of patient-centered outcomes All the patient-centered outcome responses along with the percentage(%) of the population agreed to the respective response that were recorded prior and after nonsurgical periodontal therapy (NSPT).

S. no.	Questions	Pre-procedure					Post-procedure				
		Strongly disagree	Disagree	Neutral	Agree	Strongly agree	Strongly disagree	Disagree	Neutral	Agree	Strongly agree
1	Did you notice any bleeding gums during brushing?	-	3 (7.5%)	3 (7.5%)	14 (35)	20 (50%)	18 (45%)	22 (55%)	-	-	-
2	Did you face difficulty in eating due to gum problems?	-	4 (10%)	7 (17.5%)	16 (40)	13 (32.5%)	16 (40%)	20 (50%)	-	4 (10%)	-
3	Are you satisfied with the position of the gums?	20 (50%)	15 (37.5%)	5 (12.5%)	-	-	18 (45%)	14 (35%)	-	4 (10%)	4 (10%)
4	Have you observed any sensitivity to hot or cold foods?	-	3 (7.5%)	2 (5%)	5 (12.5%)	30 (75%)	10 (25%)	10 (25%)	-	15 (37.5%)	5 (12.5%)
5	Have you avoided showing your teeth when smiling?	-	4 (10%)	7 (17.5%)	13 (32.5%)	16 (40.8%)	20 (50%)	5 (12.5%)	-	15 (37.5%)	-
6	Do you have aesthetic dissatisfaction regarding your teeth?	-	-	10 (25%)	20 (50%)	10 (25%)	27 (67.5%)	6 (15%)	-	5 (12.5%)	2 (5%)
7	Have you noticed the bad breath?	-	-	-	20 (50%)	20 (50%)	30 (75%)	10 (25%)	-	-	-
8	Do you have any problem due to food being trapped in between teeth?	-	4 (10%)	7 (17.5%)	13 (32.5%)	16 (40%)	20 (50%)	20 (50%)	-	-	-
9	Did you notice any mobility in your teeth?	24 (60%)	14 (35%)	-	2 (5%)	-	28 (70%)	12 (30%)	-	-	-
10	Have you ever faced difficulty in your work place or lost your confidence in your job?	-	6 (15%)	3 (7.5%)	17 (42.5%)	14 (35%)	12 (30%)	15 (37.5%)	1 (2.5%)	5 (12.5%)	7 (17.5%)

All 100% of patients satisfied with the reduction in bleeding gums after NSPT. About 90% of patients experienced an improvement in the functionality of their teeth in terms of chewing and biting food, following NSPT, and 10% of patients did not perceive any improvement in eating post-operatively. Preoperatively, 87.5% of patients were dissatisfied with the position of their gums, and 80% of patients observed an improvement in the position of their gums post-NSPT. About 20% of patients did not perceive any change in the position of their gums even after the therapy. Before the procedure, 87.5% of patients had sensitivity. Post-operatively, 50% of patients reported a reduction in sensitivity of their teeth. The remaining 50% still complained about sensitivity.

Regarding confidence in smiling, 73.3% of patients were not satisfied with their smile preoperatively. while 62.5% of patients were satisfied with their smile post-operatively. About 75% of the study population were aesthetically dissatisfied before treatment. Around 82.5% of patients expressed satisfaction with the appearance of their teeth after the therapy. However, 17.5% of the patients included in the study expressed dissatisfaction with the appearance of their teeth following NSPT. Also, 100% patient satisfaction was observed regarding the reduction in bad breath after the therapy. About 72.5% of patients reported food entrapment between the teeth preoperatively, and all were satisfied (100%) with reduction in food lodgment post-therapy.

Preoperatively, 95% of patients strongly agreed with the mobility of their teeth. Following the therapy after three months, mobility of the teeth was reduced by 100%. About 77.5% of patients strongly agreed with experiencing difficulty in the workplace before NSPT. Post-therapy, the percentage of patients experiencing workplace difficulty decreased to 67.5%. Regarding patient-centered outcomes, there was a statistically significant enhancement in terms of bleeding gums, eating, sensitivity, smile, aesthetics, bad breath, food entrapment, mobility, and confidence level (p < 0.05). However, no statistic significant improvement was observed in the position of the gums (p > 0.05).

## Discussion

The study evaluated the clinical parameters and patient-centered outcomes in patients who underwent NSPT in patients with generalized chronic periodontitis. All clinical parameters showed a significant reduction post-periodontal therapy, which suggests positive changes in BOP, PPD, and CAL indicating the effectiveness of NSPT [12].

The measure of patient-centered outcomes following full-mouth NSPT was measured using a customized questionnaire adopted from the DIDL questionnaire [13]. The questionnaire includes the appearance of the gums and teeth, evaluation of the patient’s comfort, difficulty in eating, bleeding gums, and the patient’s overall performance. The present study assesses the patient's perspective prior to NSPT and during the three-month review visit. This comprehensive approach not only focuses on the clinical parameters but also considers the subjective experiences and patient’s life through the assessment of patient-centered outcomes [14].

This study pioneers in assessing the impact of NSPT using a modified DIDL questionnaire. This study helps in understanding the broader implications of periodontal interventions beyond traditional clinical measures as previous studies have primarily applied the DIDL questionnaire to assess the impact of implants [15] and dental caries [16]. These studies indicate the importance of assessing patient-based outcomes in addition to the clinical parameters. This holistic approach helps in understanding the NSPT effect on the patient's life quality and provides insights for further research.

Many randomized controlled trials (RCTs) focus on PPD and CAL as primary measures of NSPT as they provide valuable objective data regarding the progression of disease and the effectiveness of interventions. However, they may not fully report the perspective of patients and the effect of treatment on their daily life [17]. Patient-centered outcomes such as pain, discomfort in teeth, and esthetic considerations help gauge the success and relevance of periodontal therapy [18].

Various studies were done to evaluate the precision of the questionnaires in dentistry. A research study investigated the relationship between removable denture prosthesis satisfaction and its impact on everyday life and personality characteristics. The measures used in the study are well-designed, validated, and reliable and enhance the validity and credibility of the findings [19]. Other studies were about the validations of two different questionnaires related to oral health-related quality of life (QOL) for children. The first validation has a questionnaire specific to Cambodian children [20], whereas the second validation was a questionnaire designed for children aged 11-14 years with diverse dental, oral, and orofacial disorders to record child perceptions [21]. Categorizing the items in the questionnaire into domains helps to organize and interpret the data more effectively. The study done by Molek et al. [22] investigated the validity and reliability of an oral health-related QOL measure in children with untreated dental caries where an extensive 25-item questionnaire was categorized into five domains.

Our study emphasized considering the NSPT effects on patients' daily lives and their subjective experiences using custom-based questionnaire along with the clinical parameters. Also, the present study explores the influence of dental interventions in improving the QOL from the perception of the patients and providing valuable insights on implications of NSPT beyond the traditional clinical metrics [23]. On the assessment of patient-centered outcomes, there was an improvement in clinical parameters, whereas regarding the patient-centered outcomes, even after three months of NSPT, almost half of the study population were not satisfied in terms of the position of the gums, sensitivity of the teeth, smile, and confidence level at workplace. On the other hand, all the study participants were satisfied with bleeding of gums, bad breath, mobility, and food entrapment.

From the study results, two important inferences can be drawn. The study suggests that the first inference was the need to identify and measure appropriate patient-centered outcomes alongside the clinical parameters following NSPT. The second inference from the study is to underscore the importance of addressing patients' primary complaints rather than focusing solely on treating the etiology of periodontal disease [24]. Despite the NSPT, if there's persistent gingival recession, further procedures such as mucogingival surgery can be considered as it aims to improve the position of the gums and enhance the aesthetic appearance of the smile. Prosthetic rehabilitation post-NSPT can be beneficial for addressing concerns related to smile aesthetics and confidence levels. This interdisciplinary approach leads to ultimate improvement in both clinical and patient-centered outcomes [25].

Limitations

The customized questionnaire used in the study might omit some details regarding patient-centered outcomes. Tailor-made questionnaires to assess patient-centered outcomes in NSPT are required as the current questionnaire was a modification of the existing questionnaire.

## Conclusions

This study concludes that significant improvement in the clinical parameters may not always directly translate to improvement in patient-centered outcomes. While NSPT can effectively address the etiology of chronic periodontitis by reducing inflammation, plaque, and probing pocket depths, it may not fully address patients' subjective experiences and concerns related to aesthetics, smile, gum position, food entrapment, and confidence levels, and these outcomes can be improved through an interdisciplinary approach.
